# Different Host Exploitation Strategies in Two Zebra Mussel-Trematode Systems: Adjustments of Host Life History Traits

**DOI:** 10.1371/journal.pone.0034029

**Published:** 2012-03-20

**Authors:** Laëtitia Minguez, Thierry Buronfosse, Laure Giambérini

**Affiliations:** 1 Université de Lorraine, Laboratoire des Interactions Ecotoxicologie, Biodiversité, Ecosystèmes (LIEBE), CNRS UMR 7146, Campus Bridoux, Metz, France; 2 Université de Lyon, Laboratoire d'endocrinologie, Ecole Nationale Vétérinaire de Lyon, Marcy l'Etoile, France; Kansas State University, United States of America

## Abstract

The zebra mussel is the intermediate host for two digenean trematodes, *Phyllodistomum folium* and *Bucephalus polymorphus*, infecting gills and the gonad respectively. Many gray areas exist relating to the host physiological disturbances associated with these infections, and the strategies used by these parasites to exploit their host without killing it. The aim of this study was to examine the host exploitation strategies of these trematodes and the associated host physiological disturbances. We hypothesized that these two parasite species, by infecting two different organs (gills or gonads), do not induce the same physiological changes. Four cellular responses (lysosomal and peroxisomal defence systems, lipidic peroxidation and lipidic reserves) in the host digestive gland were studied by histochemistry and stereology, as well as the energetic reserves available in gonads. Moreover, two indices were calculated related to the reproductive status and the physiological condition of the organisms. Both parasites induced adjustments of zebra mussel life history traits. The host-exploitation strategy adopted by *P. folium* would occur during a short-term period due to gill deformation, and could be defined as “virulent.” Moreover, this parasite had significant host gender-dependent effects: infected males displayed a slowed-down metabolism and energetic reserves more allocated to growth, whereas females displayed better defences and would allocate more energy to reproduction and maintenance. In contrast, *B. polymorphus* would be a more “prudent” parasite, exploiting its host during a long-term period through the consumption of reserves allocated to reproduction.

## Introduction

By definition a parasite is an organism that feeds, hides itself or breeds at the expense of its host [1]. This host-parasite interaction has several negative effects for the host which deprives it of resources or induces some tissue damages [2]. The host physiology is therefore modified by the presence of parasites. With this in mind, in the framework of environmental pollution risk assessment, parasitism can be a confounding factor interacting with other environmental factors [3]–[10]. Indeed, the physiological modifications observed following the infection can lead to false-positive and false-negative conclusions [6]. Parasites inevitably affect the life-history traits of their hosts (i.e. survival, growth and reproduction) [2]. Likewise, parasitized individuals can be more tolerant [11] or more susceptible to other environmental factors (e.g. pollution, hypoxia) [12]–[15]. Adjustments of growth have also been well documented particularly in snail-trematode systems, with infected organisms which often displayed greater sizes. This phenomenon was called gigantism [16]–[18]. Finally, the investment in reproduction (“fecundity compensation”) can be modified followed parasitism. For example, Minchela & Loverde (1981) [19] have observed a higher egg production in *Biomphalaria glabrata* snails infected by *Schistosoma mansoni*. However, only a few studies were concerned with freshwater host-parasite systems [5], [8], [11], [20].

Our research in environmental parasitology focuses on host-parasite interactions with the freshwater bivalve, *Dreissena polymorpha* (Pallas, 1771) [8], [21]. The zebra mussel is an invasive species coming from the Caspian area which has spread widely throughout Europe and North America [22]–[23]. Like all bivalves, *D. polymorpha*, commonly called zebra mussel, has physiological and behavioral characteristics (e.g. sedentarity, filtration of large water volume, bioaccumulation of xenobiotics) that make them good sentinel organisms in water quality assessment [24]–[26]. These mussels have also been documented to have a variety of parasites and symbionts. Over 45 species have been reported to use *D. polymorpha* as intermediate or as unique host in their life cycle [27]–[30]. Among these endosymbionts, 7 genera of parasitic trematodes have been described [27], [31]. This paper focuses on infections of two trematode species, *Phyllodistomum folium* and *Bucephalus polymorphus* in *D. polymorpha* and is a further contribution towards understanding the endoparasites of this mussel.

In the life cycles of these two trematodes, *D. polymorpha* is the first intermediate host [27]. The two life cycles begin with the development of the miracidium (i.e. the earliest larval stage) which penetrate either gill demibranch for *P. folium* or gonad area for *B. polymorphus*. The miracidium then develops into the sporocyst and cercariae grow within this last. From this step, the two trematode species have more specific development. In *P. folium*, mature sporocysts containing metacercariae are shed from the gills and are carried by the current until they are consumed by fish (final host). In contrast, *B. polymorphus* needs a second intermediate host in its life cycle, more frequently cyprinid fishes in which cercariae develop into metacercariae. The final hosts are carnivorous fishes, infected with metacercariae while eating infected cyprinids. For both parasites metacercariae develop into adults, become sexually mature, and after fertilisation the life cycle starts again [27], [32].

In complex parasite life cycles, the intermediate host plays not only the role of vessels for parasite transmission to the next host, but also the role of food providing sufficient energetic resources for parasite development [33]–[34]. These host-parasite relationships are the result of a trade-off between the benefits of resource consumption and the costs of reduced host viability [34]. Several host exploitations are possible: a ‘prudent’ strategy in which the parasite keeps its host alive for a long period without noticeable physiological damages and produces few parasites per unit time or a ‘virulent’ strategy in which the parasite development is fast and the host is killed quickly [35]–[36]. As said by Jokela *et al.* (1993) [37], a successful ‘parasite strategy’ is one where the host is not killed before it has fulfilled its function from the parasite point of view.

The aim of this study was to examine the host exploitation strategies of *P. folium* and *B. polymorphus*, and adjustments of *D. polymorpha* life history traits following infections. We hypothesized that these trematodes, by infecting two different organs, do not interact in the same way with the host. To test it, several biological responses were studied: four cellular responses in the digestive gland related to the general physiological status of zebra mussels, the energetic reserves available in gonads (i.e. protein, triglyceride and glycogen levels), the shell lengths and the gonad maturity.

## Materials and Methods

### Sample preparation

Zebra mussels were collected in two sites in France: Troussey on the Meuse River (48°42′13.89″N, 5°42′02.99″E), on March 2009, to look for organisms infected by *P. folium*, and Langon on the Vilaine River (47°42′56.95″N, 1°50′13.41″W), on August 2009 for *B. polymorphus*. At both public and non protected locations, no permissions were required for collecting zebra mussels considered as invasive species. The shell length range of zebra mussels from Troussey was 23–37 mm and 15–21 mm for zebra mussels from Langon. A sampling effort was made to find enough parasitized zebra mussels, due to the low prevalence rates of the two trematode species. The mussel valves were opened taking care not to break it, and the gills or gonads were examined under a stereogical microscope for the presence of infection by *P. folium* or *B. polymorphus*, respectively. The prevalence rates of *P. folium* and *B. polymorphus* in *D. polymorpha* (n = 564 and n = 905, respectively) was determined as the percentage of visually infected organisms.

After thoroughly cleaning all shells for any surface deposits or epibionts, the maximum length (L), height (h) and width (w) were measured with a calliper rule to the nearest 0.01 mm. The soft tissues were then removed and excess body fluid absorbed with absorbent paper. The soft tissue wet weights (m) and the shell weights were obtained on a Pioneer PA214CM balance to the nearest 0.1 mg. A condition index (CI) expressed in g.cm^3^, was calculated as follows: CI = m/ (L×h×w).

For each organism, a part of the gonad (also weighed) and the digestive gland were then excised and served to quantify energetic reserves and cellular responses, respectively (see below). All the remaining tissues were used for the parasite inventory.

### Parasite inventory

Trematode-infected and –uninfected organisms were identified after dissections. The procedure for microparasite detection by classical histological techniques is described by Minguez *et al.* (2009) [8]. Only non-infected organisms (i.e. absence of micro- and macroparasites), randomly chosen and those infected only by *P. folium* or *B. polymorphus* were kept for biomarker analysis. The host sex was also taken into account except for zebra mussels infected by *B. polymorphus* (i.e. castration). In each experimental group, zebra mussels displayed the same mean trematode load level. The table 1 indicates the number of organisms used in each experimental group.

**Table 1:** Number of zebra mussels in each experimental group used for the analysis of biological response.

		N	N
*Phyllodistomum folium*:			*Bucephalus polymorphus*:	
Males	Non-Infected	16	Non-Infected	12 males+12 females
	Infected	16	Infected	24
Females	Non-Infected	9		
	Infected	9		

### Determination of gonadal index

Gonad maturity was assessed by microscopic observation of the slides, through the determination of a mean gonadal index (GI) for each experimental group [38]. Mussels were classified in one of six successive stages of gonad maturation, common or not to both sexes: an apparent sexual rest (stage 0), gametogenesis initiation (stage Ia), early gametogenesis (stage Ib for males and stage IaPrS for females), advanced gametogenesis (stage II for males and stage IaS for females), sexual maturity (stage III for males and stage IaPostS for females) and spawning (stageE). An arbitrary score from 0 to 5 was attributed to each stage, and the following formula was used to calculate gonadal index: GI = (Σ n_i_.s_i_)/N where n_i_ is the number of individuals in each stage, s_i_ the score of the stage and N the total number of individuals.

### Histochemistry and stereology on the digestive gland

The removed digestive glands were prepared as described in Giambérini & Cajaraville (2005) [39] and used to measure four cellular responses: the structural changes of the lysosomal system, the peroxisomal catalase activity, and the accumulation of neutral lipids and lipofuscin granules. The digestive lysosomal system was located by the revelation of β-glucuronidase activity in unfixed cryostat sections according to Giambérini & Cajaraville (2005) [39]. Unsaturated neutral lipids were demonstrated by oil red O staining [40], and lipofuscin granules were stained by the Schmorl reaction [41]. The histochemical revelation of the peroxisomal catalase in unfixed cryostat sections was adapted from Cajaraville *et al.* (1993) [42], as described in Guerlet *et al.* (2006) [43]. These cellular biomarkers were quantified on digestive tissue sections (8 µm) by image analysis (Cell*, Olympus) using a Sony DP 50 color video camera connected to an Olympus BX 41 microscope with a 100× objective. Five fields of view were randomly analysed on one section per individual. Areas not belonging to digestive tissues were discarded from analysis. The stereological parameters shown herein to simplify the dataset are the volume density of the lysosomal and peroxysomal system, and the surface densities of neutral lipid droplets and lipofuscin granules (Vv_L_ = V_L_/V_C_; Vv_P_ = V_P_/V_C_; Sv_NL_ = S_NL_/V_C_; Sv_LF_ = S_LF_/V_C_, where C = digestive cell cytoplasm, L = lysosomes, P = peroxisomes, NL = neutral lipids, LF = lipofuscin, S = surface, V = volume).

### Energetic reserves in gonads

The levels of proteins, triglycerides and glycogen were measured on excised gonads. Samples were homogenized in 200 µL PBS 1× and energetic reserves directly measured using a robot commonly used for the clinical analyses, Konelab® (Thermo Fisher Scientific, Cergy Pontoise). Measurements were based on colorimetric methods [44]. Briefly, the glycerol obtained after the hydrolysis of triglycerides by a lipase, was measured at 510 nm. The bond between proteins and red pyrogallol-molybdate complexes was measured at 600 nm. The glycogen was extracted and digested by an amiloglucosidase (CAS 9032-08-0, Sigma Aldrich, France). Then, glucose was oxidized by a glucose-oxydase leading to a chromogen whose concentration was measured at 510 nm. Finally, glycogen concentrations were calculated using oyster glycogen as calibrating control (CAS 9005-683-8, Sigma-Aldrich, France).

### Data analysis

All statistical analyses were carried out using Statistica software version 7.1. (Statsoft, USA). Significant differences between experimental groups were studied using t-tests. The analyses were performed after testing for normality and homogeneity of the data. In order to achieve homoscedasticity (assessed by Brown–Forsythe test), all the cellular parameters were log-transformed before the analysis. P-values less than 0.05 were considered statistically significant.

## Results

### *Phyllodistomum folium*

**Figure 1:**
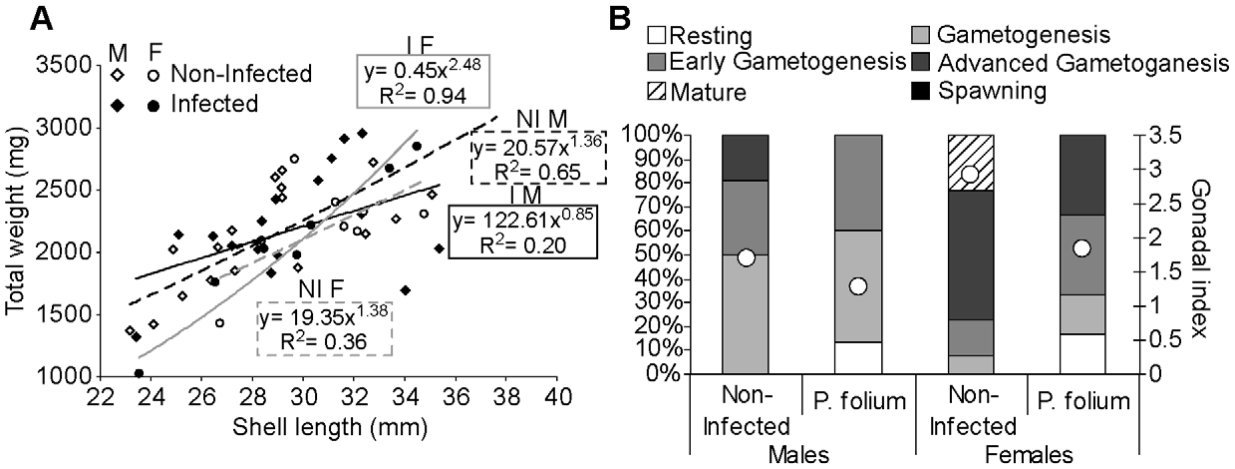
Allometric relationships and Reproductive status of zebra mussels infected or not by *P. folium*. Allometric relationships of shell length (cm) and total weight (i.e. sum of shell and wet weights) (g) for male and females zebra mussels infected or not by *P. folium* (*A*). Full points: infected organisms (I), Empty points: non-infected organisms (NI). Solid line: allometric relationships for infected individuals (Black: males (M), Grey: females (F)), Dashed line: allometric relationships for the non-infected (Black: males (M), Grey: females (F)). Percentages of mussels at each gamete developmental stage and gonadal index values (white dots) (*B*), in function of the mussel gender and the infection status.

**Figure 2:**
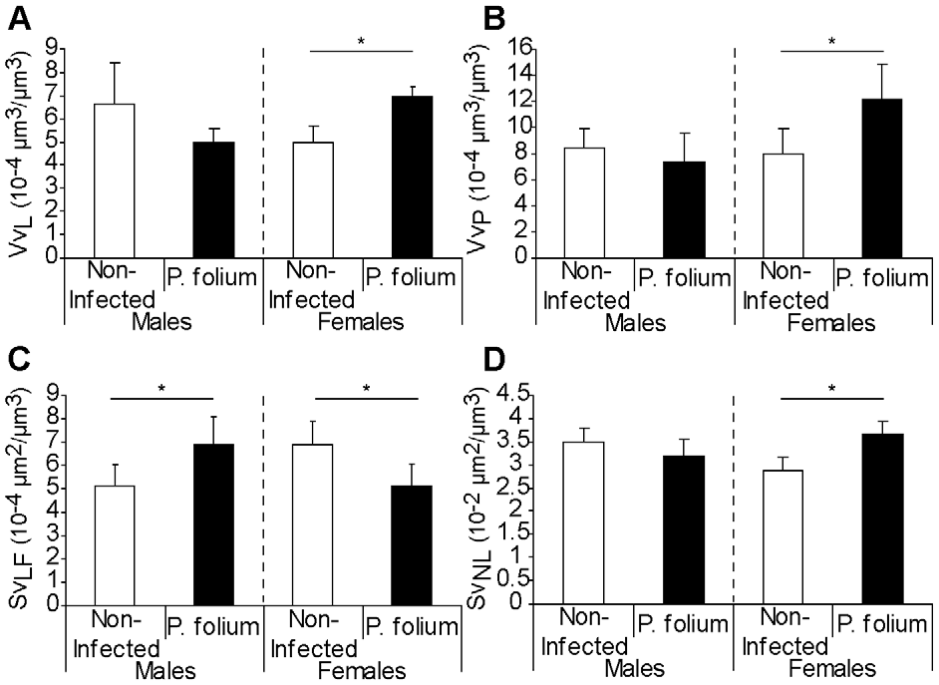
Cellular responses in the digestive gland of zebra mussels infected or not by *P. folium*. The four cellular responses measured in the digestive gland of zebra mussels, males and females, infected or not by *P. folium* (mean values ± S.D.): (*A*) the structural changes of the lysosomal and (*B*) peroxisomal systems (volume densities), (*C*) the accumulation of lipofuscin granules and (*D*) neutral lipids (surface densities). The effect of infection was evaluated for each sex separately by a t-test and a significant difference was indicated by an asterisk (p<0.05).

**Figure 3:**
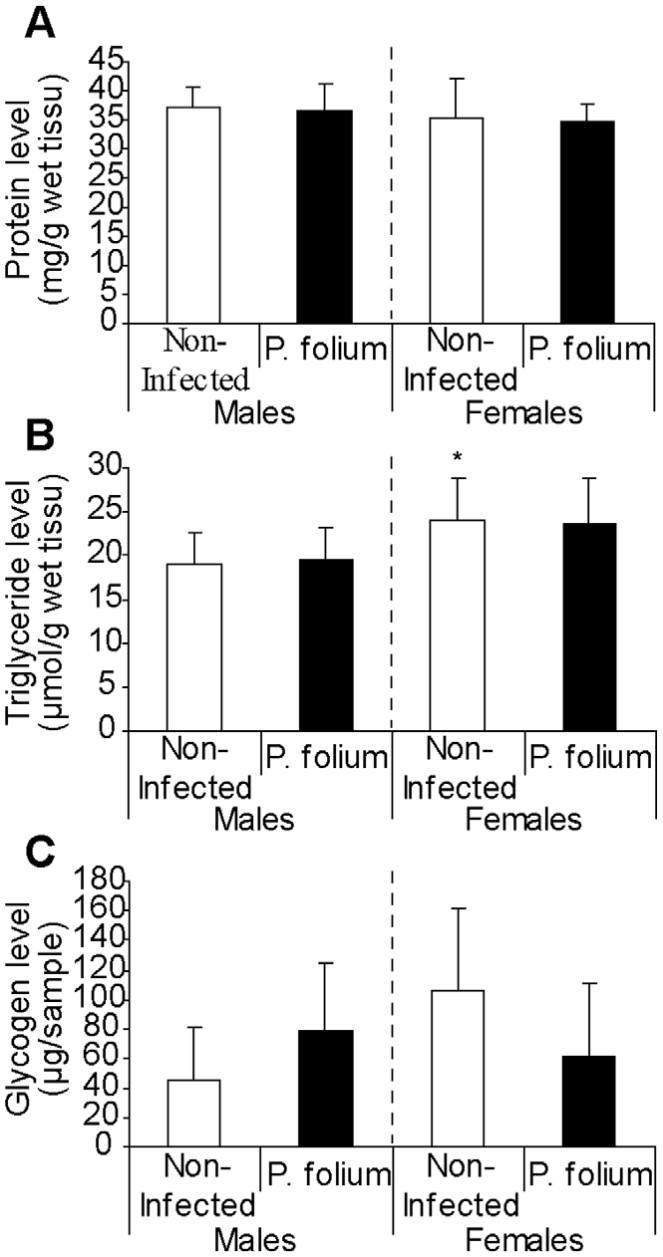
Gonadal energetic reserves of zebra mussels infected or not by *P. folium*. Energetic reserves available in gonads of zebra mussels, males and females, infected or not by *P. folium* (mean values ± S.D.): (*A*) Protein, (*B*) Triglyceride and, (*C*) Glycogen levels. The asterisk means significant difference between non-infected males and females (p<0.05).

Twenty five (4.4%) of the 564 dissected zebra mussels from Troussey (Meuse River) were parasitized by *P. folium*, with two times more infected males. The growth and the reproductive status of non-infected and infected mussels, males and females, were shown on the figure 1 (A and B). Infection tended to have the opposite effect on males and females. The first would grow up faster than they would gain weight (Figure 1A). However, the infection had no significant effects on condition indices of males and females mussels (data not shown). *P. folium* was always associated with a lower gonadal index in zebra mussels (Figure 1B). Infected organisms displayed a delay in gonad development (i.e. early stages of gonad maturity). It should also be noted that females were consistently more advanced in their reproduction cycle. Looking at the biological responses assessed in the digestive gland, the infection seemed to have different effects according to the host sex (Figure 2). The digestive lysosomal system tended to be less developed in parasitized males compared with non-infected ones whereas females displayed a completely opposite response with a significant activation of the system in infected mussels (t-test, p = 0.019) (Figure 2A) with bigger and more numerous lysosomes. Moreover, *P. folium* induced a higher peroxisomal system development only in females (t-test, p = 0.046). No significant differences were observed in males (Figure 2B). As in the lysosomal system, males and females displayed opposite responses for their lipofuscin contents. Indeed, the infection was associated with higher levels of lipofuscin granules in males (t-test, p = 0.019) but less cellular damages in females (t-test, p = 0.049) (Figure 2C). A significant accumulation of neutral lipids in the digestive gland was observed in infected females (t-test, p = 0.022) whereas males displayed the same neutral lipid contents whatever their infection status (Figure 2D). The energetic reserve levels quantified in gonads were shown in Figure 3. The infection by *P. folium* seemed to affect neither the protein nor the triglyceride levels (Figure 3A and B). However, females displayed significantly higher lipid contents in gonads than males, whatever the infection status (t-test, p = 0.048). Concerning the glycogen levels no significant differences were observed due to an important intra-group variability, but it should be noted a tendency in infected males to have higher glycogen contents compared with non-infected ones, unlikely infected females for which the opposite was observed (Figure 3C).

### *Bucephalus polymorphus*

**Figure 4:**
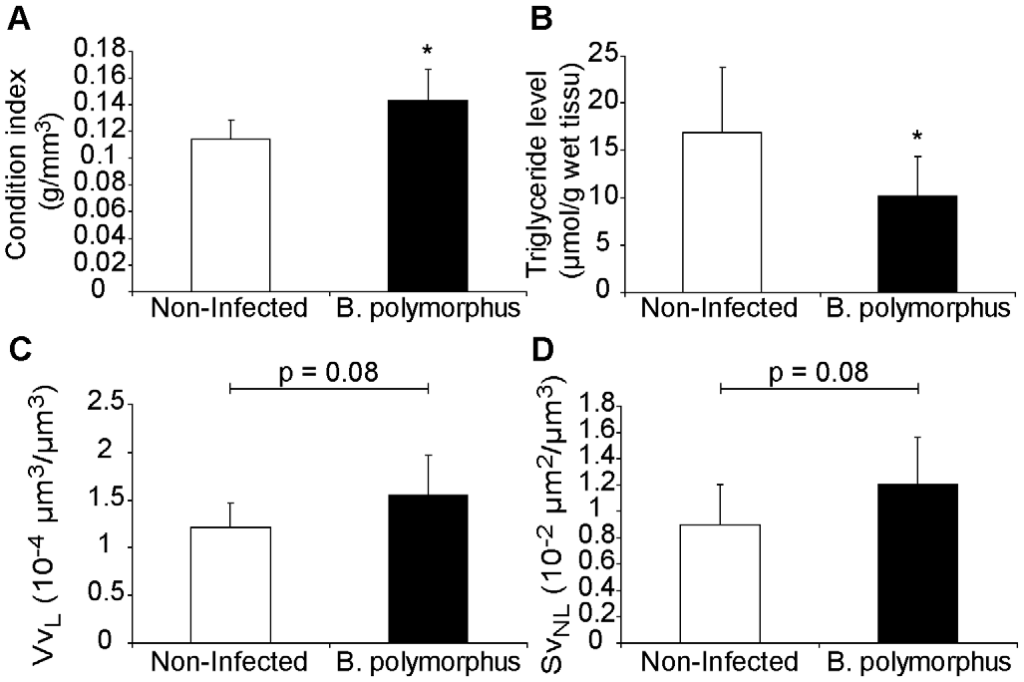
Effects of the trematode *Bucephalus polymorphus* on four physiological parameters of the zebra mussel (mean values ± S.D.). (A) Condition index, (B) Level of triglycerides in the gonads, (C) the structural changes of the lysosomale (volume density), and (D) the neutral lipid content in the digestive gland (surface density). Significant differences were indicated by an asterisk (t-test, p<0.05).

The prevalence of the parasite *B. polymorphus* in *D. polymorpha* from Langon (Vilaine River) was 1.9%. The infection was relatively well advanced since gonads were totally replaced by parasite sporocysts, involving the sterility of the host. The other organs were not infected. We can note that non-infected zebra mussels were ready for gamete emission (gonadal index = 4.84). Infected organisms displayed significantly a higher condition index (t-test, p<0.05) (Figure 4A) and less triglyceride reserves in gonads than non-infected mussels (t-test, p<0.001) (Figure 4B). *B. polymorphus* tended to induce a more developed digestive lysosomal system and a higher accumulation of neutral lipids in the digestive gland (t-test, p = 0.08) (Figure 4C and D). For the other cellular responses and energetic reserves assessed (i.e. the structural changes of the peroxysomal system, the accumulation of lipofuscin granules, the protein and the glycogen levels), no significant differences were observed according to the infection status (data not shown).

## Discussion

Most bivalve species harbour a variety of parasites, but little is known about their interactions with host physiology [45]. This is especially true when dealing with freshwater bivalves with no economic interest. In this context, trematode parasites represent an interesting model to study host-parasite interactions and host exploitation strategies, since some are highly pathogenic whereas others are less virulent with longer infectious periods. Infections result from a trade-off between the use of host energy by the parasite for its own development and the maintenance of the host life [34], [36], [46]. Concerning the zebra mussel, many grey areas exist in the physiological disturbances related to parasitism by trematode species. The present study brings additional information on *D. polymorpha-P. folium* and *D. polymorpha-B. polymorphus* systems. This research becomes integrated in the framework of environmental parasitology with useful data not only for parasitologists in terms of host exploitation strategies but also for ecotoxicologists using zebra mussels as test organisms since parasites can be a source of data distortion.

The first parasite investigated was the digenean trematode *P. folium* observed within branchial sinuses. The effect of this parasite on the zebra mussel physiology seemed mainly to be host sex-dependent, with males and females often responding in an opposite manner. Infected males showed evidence of cell alteration with higher levels of lipofuscin granules compared with non-infected males. The gill deformations observed in infected individuals could lead to the activation of the immune system with inflammatory reactions [31]. The immune responses especially involve the production of highly reactive oxygen species (ROS) [47]–[48]. Thus, this oxidative burst could cause the cellular damaged observed in infected males. The infection was also linked with a catalase activity which tended to be lower. The antioxidant system would be unable to overcome the generation of ROS. The parasite tended also to induce a less developed digestive lysosomal system in males. These observations suggest a slowdown of the infected-male metabolism, particularly in terms of cellular defence system failure. These features involved an increased sensibility to environmental conditions and could explain the higher infection prevalence observed in males. In contrast, infected females seemed to exhibit better defences with a more developed lysosomal and peroxisomal system which would fully play its role of antioxidant system with few signs of cellular alterations (i.e. less lipofuscin content). Concerning the energetic content available in parasitized mussels, differences were also observed between males and females, suggesting a different use of the energy stock. All organisms have a finite energy content for growth, reproduction and maintenance (Cody, 1966 cited in [37]). Because of this, growth and reproduction cannot both be maximised simultaneously and, in stressful conditions, they exert conflicting demands on organisms [49]. Parasitism may represent precisely a stress for organisms and to date, no study has focused on strategies of energetic reserves use in *D. polymorpha* – trematode systems. Herein, females when infected, displayed higher levels of neutral lipids in the digestive gland but tend to have lower glycogen content in gonads and smaller shells. These results suggest a re-allocation of energy to reproduction rather than growth, since during gametogenesis, carbohydrates and particularly glycogen are converted into lipids in gonads and stored [38], [50]–[52]. The higher gonadal index measured in females could be precisely linked with this reserve-exploitation strategy. Nevertheless, the slight delay in gonad maturity highlighted in infected females could be explained by the use of an important part of the available energy to defence activities (e.g. higher development of some cellular compartments). A reduced gonadal tissue has yet been reported in the system *D. polymorpha*-*P. macrocotyle* without preventing the gamete production [27], [53]. Stoeckman & Garton (2001) [49] have shown that zebra mussels are flexible in energy allocation: in conditions when survival is assured zebra mussels decreased their reproductive effort whereas in stress conditions, the reproductive effort was increased. In this sense, *P. folium* may impair the survival of *D. polymorpha* due to gill deformations and disturbances of filtration activities. Kraak & Davids (1991) [25] have observed deleterious effects of another trematode belonging to the same genus (i.e. *Phyllodistomum macrocotyle*) on zebra mussels, like a dramatic decrease of their total dry weight associated with higher accumulation of metals in tissues. Infected males displayed another strategy, maybe allocating more energy to growth. Here, shell lengths tended to be higher in infected males compared with non-infected congeners. In snail-trematode systems this kind of phenotypic modification has already been described as gigantism, since infected organisms often attained larger sizes than uninfected congeners [16]–[18]. Here, we cannot really talk about gigantism in view of similar shell length ranges between infected and non-infected males; nevertheless the infection seemed to induce a modification of this life-history trait in this sense. This phenomenon is interpreted as a side-effect of castration, with the energy normally allocated to reproduction used for growth [18]. However, *P. folium* is not a castrator parasite and so, may rather fall into the alternative interpretation: this greater shell length would be a host adaptation allowing the mussel to outlive the infection with the possibility of achieving reproductive status thereafter [16], [18], [54]. Infected males tended to display higher levels of glycogen in gonads which represents one of the main organs of glycogen storage in bivalves [37]. The increase in glycogen content could be an indirect consequence of the increasing energetic demands experienced by infected males, as previously noted by Plaistow *et al.* (2001) [55] in the system *Gammarus pulex* - *Pomphorhynchus laevis*.

The other studied digenean trematode was *B. polymorphus*, a castrating parasite host-specific to *Dreissena* [27], [31]. We found that for the same shell length range, infected zebra mussels displayed higher condition indices than non-infected conspecifics. This would be simply due to the weight of sporocyst branches occupying the entire gonad, which would be greater than gamete weight, and not really because of a better health status. Castrated mussels tended also to display a more developed digestive lysosomal system with enlarged lysosomes. This is in agreement with another study on the white furrow shell (*Abra abra*) infected by *Bucephaloides gracilescens* (Trematoda), a castrator parasite too, where infection involved a higher lysosomal activity [56]. Herein, we can associate this lysosomal response with the highest level of neutral lipids in the digestive gland. The development of *B. polymorphus* would require more energy leading to the increase of food intake by zebra mussels. The lysosomal system precisely occupies a central and crucial role in intra- and extracellular food degradation [57]–[58] and would be thus activated in an infected organism. The increased food intake would also involve higher neutral lipid storage in the digestive gland since these triglycerides are known to have an alimentary origin [43], [59]–[60]. They principally represent a nutritive content intended for gametogenesis and secondary energetic resources for the growth [40], [43]. The proportion of assimilated energy allocated to reproduction is relatively high in molluscs [61] and so, by infecting gonad and synchronizing the production of cercariae with the production of gametes in uninfected zebra mussels, the trematode can efficiently use the host's reproductive energy [37], [62]–[63]. This strategy is considered as ideal for reducing the effect of infection on host survival, whilst still using host reserves [64]. It is supposed that once infected, mussel gonads will annually produce cercariae instead of gametes for the rest of its life [27], [31]. Thus, *B. polymorphus* seemed not to be lethal for *D. polymorpha* and in a sense, as Kuris (1974) [65] highlighted parasitic castrators have some similarities with parasitoid since for example the first cause the reproductive death of only one host and the other consumes only one host during its lifetime. Both use host energy until the end of their development. We found also that infected mussels displayed a lower triglyceride level in the gonad. However, Jokela *et al.* (1993) [37] found the opposite result in infected clams (*Anadonta piscinalis*) and supposed that the trematode sporocyst must contain a lot of fat, all the more that Taskinen *et al.* (1991) [66] showed that cercariae use lipids as an energy base to swim after release. In our investigation, if the difference in triglyceride levels in gonad is so significant between infected and uninfected organisms, it could be due to the high level of lipids measured in uninfected females (i.e. ovocytes rich in lipids) whereas uninfected males displayed on average the same lipid content as infected mussels.

In this study, we examined zebra mussel physiological modifications associated with infections by two trematodes using *D. polymorpha* as first intermediate host. *P. folium* and *B. polymorphus* are localised in gills and gonads respectively, and seemed to affect digestive cellular responses but to various degrees. Both parasites induced host-life history trait adjustments. *P. folium* had significant host gender-dependent effects. Infected males displayed a slowed-down metabolism and energetic reserves more allocated to growth, whereas females displayed better defenses and would allocate more energy to reproduction and maintenance. *B. polymorphus* induced fewer changes in the tested biological responses. According to the literature [28], [31], [35]–[36] and to the host life history traits measured in the present study in terms of maintenance (defence systems, energetic reserves), reproduction (gonadal index) and shell morphology/growth (condition index), we could hypothesize that *P. folium* would experience short-term host exploitation due to its pathogenicity and would adopt a ‘virulent strategy’. In contrast, *B. polymorphus* would use zebra mussels more during long-term periods, with release of cercaria instead of gametes during the spawning period and would follow a more ‘prudent’ strategy suggesting that the gonad is less critical for survival of the host in comparison with the gills. This investigation brings necessary information to better understand these *D. polymorpha* – trematode systems but further studies will be necessary to confirm these results at both parasite level (e.g. number of cercaria released per unit times) and host level (e.g. survival).
